# Supplementary material for: “Mothers will be lucky if utmost receive a single scheduled postnatal home visit”: An exploratory qualitative study, Northern Ethiopia
Source: PLoS One. 2022 Mar 30;17(3):e0265301. doi: 10.1371/journal.pone.0265301 (PMC8967047; doi:10.1371/journal.pone.0265301)
Supplement: S4 File — (DOCX) [file pone.0265301.s004.docx]

**MEKELLE UNIVERSITY, COLLEGE OF HEALTH SCIENCES**

**SCHOOL OF PUBLIC HEALTH**

**YEMANE BERHANE**

**Research objective:**  To explore the barriers and facilitators of PNHV by HEWs in rural Tigray, northern Ethiopia.

**KIIs: Experts and supervisors**

Thank you for completing the informed consent form and for taking the time to speak with me today. I have several questions to ask you that I have prepared in advance. I will turn on the tape recorder now. If you have any additional questions or comments as we do the interview, please feel free to share them with me.

**Interview details**

1. District: _________________________
2. Interviewer name/ID:___________________
3. KII code__________
4. Date of Interview_________________________
5. Interview start time:_________________________
6. Interview end time: _________________________
7. Tape Recording number:________________

**Part 1**: Socio-demographic characteristics

| KII No. | Age | sex | Occupation | Religion | Marital status | Educational level | Parity | Experience as supervisor | Total work experience | Remark |
| --- | --- | --- | --- | --- | --- | --- | --- | --- | --- | --- |
|  |  |  |  |  |  |  |  |  |  |  |

**Part 2**: How do you explain the provision of PNHV at home from your experience?

**Proving: -** Does the service provided at home?

-When do you think the schedule of PNHV/ what are the contents of PNHV?

-What activities do you think conducted during postnatal home visit (type of support for the mother and newborn, type of Materials and supplies for PNHV used?)

**Part 3**: what do you think is the barriers and facilitators of PNHV?

**Proving**: (Motivation of HEWs, incentives, Linkage, Transport access and distance, Topography, Training and supportive supervision, Human resource, Materials and equipment, Government concern/priority, leadership and governance, community support, workload, Perceptions of HEW to wards PNC, willingness to receive PNHVs and perceptions of mothers towards the importance of the services**)**

**Part 4**: How do you explain the role of HEWs in conducting PNHV and solving postnatal complications of the mother and newborns at home?

**Proving**:-Provision of quality and scheduled PNC with full contents

-Solving Postnatal challenges on time

-Linkage with HEWs

**Part 5**: What is your opinion to improve PNHV coverage (by HEWs, health centers, district health office, regional health bureau, and the community?)

**Thank you.**
