# Supplementary material for: “Mothers will be lucky if utmost receive a single scheduled postnatal home visit”: An exploratory qualitative study, Northern Ethiopia
Source: PLoS One. 2022 Mar 30;17(3):e0265301. doi: 10.1371/journal.pone.0265301 (PMC8967047; doi:10.1371/journal.pone.0265301)
Supplement: Attachment — Submitted filename: Response to Reviewers.docx [file pone.0265301.s007.docx]

**Responses to Reviewers’ and Editors**

We wish to express our appreciation to the reviewers for their comments, which have helped us to improve our manuscript. We have thoroughly revised our manuscript titled **“Mothers will be lucky if utmost receive a single scheduled postnatal home visit”: an exploratory qualitative study, Northern Ethiopia.** We have incorporated and highlighted the changes in the revised manuscript based on the suggestions made by the editor and reviewers.

Yemane Berhane Tesfau

Below follows a point-by-point response to the reviewers’ comments.

**Responses to editors**

Comments: Thank you for providing additional details regarding the restrictions on data sharing. At this time, please confirm that your submission contains your "minimal data set", which PLOS defines as consisting of the data set used to reach the conclusions drawn in the manuscript with related metadata and methods, and any additional data required to replicate the reported study findings in their entirety. This includes:
1) The values behind the means, standard deviations and other measures reported;
2) The values used to build graphs;
3) The points extracted from images for analysis.
If so, we will update your Data Availability Statement to reflect this information.
If the minimal data is not present, and there are ethical or legal restrictions on sharing a de-identified minimal data set, please explain them in detail (e.g., data contain potentially sensitive information, etc.) and who has imposed them (e.g., an ethics committee). Please also provide contact information for a data access committee, ethics committee, or other institutional body to which data requests may be sent.

**Response**: Thank you very much for the comments and concerns you raised. The values behind the means, standard deviations and other measures were reported in the participants’ demographic characteristics under the result section of the manuscript. Data cannot be shared publicly because data contains potentially sensitive information and some of the informants could be identified just by the story they tell. All relevant data are within the manuscript and its Supporting Information files. Raw data cannot be shared publicly because they contain potentially sensitive and identifiable information. We have not used any graph and images for analysis. However, if any body needs details of the data, he/she can contact the first author.

**Responses to comments from reviewer #1**

Overall comment: The manuscript is well written and appreciates the hard work involved in the conduct of the study.

- Thank you very much for appreciating and acknowledging our work.

**Comment 1**: Please mention the research team and their credentials, who conducted qualitative interviews and FGDs, experience, and training.

**Response**: Thank you for pointing out this and we have incorporated it in the data collection section.

**Comment 2**: Study design should include

**Response:** We agree with your comment and we have included:

- The methodological orientation: exploratory qualitative research using thematic analysis
- Participant selection: sampling, method of approach, and sample size

-we have no any refusal to participation in the study

- In the data collection section mention any repeat interview: we did not have any repeat interview

**Comment 3**: Data analysis: how member checking was conducted

**Response**: we agree with you and we have re-written as:

To verify our interpretation as sound, we the authors checked the document and tried to brief about the contents to a sample of the participants in the study districts.

**Results section:**

**Comment 4**: suggestion to reduce the quotable quotes

**Response**: we agree with you and we tried to reduce and summarize the quotes.

Discussion

**Comment 5**: Editing is required

**Response**: we agree with your constructive comments and we did it.

**Comment 6:** What is new in this study? Why should this article be cited?

**Response**: Thank you for your constructive comments and we revised it to address these issues. Except for the sake of political agenda, attention was not given to postnatal care by the health care authorities in northern Ethiopia. The focus of healthcare authorities in northern Ethiopia were on other maternal and child health care services like coverage of 4^th^ ANC, improving facility delivery, and child immunization; despite evidences of maternal and newborn deaths at home during the postnatal period.

**Comment 7**: How the barriers (home visits) in Africa or your region is different from other low and middle- income countries?

**Response**: Thank you we agree with you. And we included in the discussion section.

**Comment 8**: What significant steps were taken by the other African countries or low and middle-income countries to address barriers?

**Response**: Thank you for your insight. We included this in the introduction section of the manuscript.

**Comment 9**: How the findings could be used: application/translation and way forward. Recommendation to the government to address the barriers

**Response:** thank you for your constructive insight, and we incorporated in the conclusion part.

**Responses to comments from Reviewer #2**

**Comment 1:** The entire manuscript requires extensive English language copy editing.

**Response:** Thank you very much and we did it with language expert.

**Comment 2:** I recommended you to reduce word count

**Response:** Thank you and we agree with you, especially in the result section.

**Comment 3:** Please revise the “introduction” just by focused on your topic.

**Response:** We absolutely agree with your concern and we tried to re-write focusing on the topic.

**Comment 4:** As you sometimes used the term HEW and sometimes CHW, are they interchangeable?

**Response:** Thank you for your observation, we can use interchangeable, however, in Ethiopia HEWs are government employed community health workers. In other countries the CHWs might be government employed or volunteers. There could be also difference in level of certification/education.

**Comment 5:** The topic should be clarified for “barriers and facilitators of scheduled postnatal home visit”

**Response:** Thank you for your suggestion and we appreciate your concern. The authors’ thinks scheduled PNHV in this study area does almost not exist. Thus, this topic expresses beyond barriers and facilitators so that healthcare authorities and concerned government bodies could give emphasis for its implementation**.**

**Comment 6:** Most of the sentences in “Study settings” are not mandatory, also not clear. And your justification in selecting the study setting does not much scientifically sound.

**Response:** Thank you very much and we agree with you.

**Comment 7:** The sample size lack theoretical ground.

**Response:** Thank you for your insight and were revised.

**Comment 8:** Some of the sentences in “data analysis” section still talk about data collection.

**Response:** yes it seems data collection but it is to mean about checking the data quality. Any way we revised it.

**Comment 9:** The manuscript does not follow the recommended reporting format for qualitative study.

**Response:** We have followed the **PLOS ONE guideline** Consolidated criteria for reporting qualitative research (**COREQ**), though there are different reporting formats for qualitative research

**Comment 10:** In “Results” direct quotations are too difficult to understand.

**Response:** Thank you for your comment and we have rephrased the quotes and tried to reduce the word counts

**Comment 11:** Please provide clear discussion concentrating on your findings.

**Response:** Thank you for your comment and agree with you.

**Comment 12:** Please sufficiently address the limitations of your study

**Response:** thank you and we addressed it.

We again thank the editors and reviewers for their insightful and constructive comments.
