# Supplementary material for: “Mothers will be lucky if utmost receive a single scheduled postnatal home visit”: An exploratory qualitative study, Northern Ethiopia
Source: PLoS One. 2022 Mar 30;17(3):e0265301. doi: 10.1371/journal.pone.0265301 (PMC8967047; doi:10.1371/journal.pone.0265301)
Supplement: Attachment — Submitted filename: Response to Reviewers.docx [file pone.0265301.s008.docx]

**Responses to Reviewers’ and Editors**

We wish to express our appreciation to the reviewers for their comments, which have helped us to improve our manuscript. We have thoroughly revised our manuscript titled **“Mothers will be lucky if utmost receive a single scheduled postnatal home visit”: an exploratory qualitative study, Northern Ethiopia.** We have incorporated and highlighted the changes in the revised manuscript based on the suggestions made by the editor and reviewers.

Yemane Berhane Tesfau

Below follows a point-by-point response to the editor’s and reviewers’ comments.

**Responses to editors**

**Comments**: Please review your reference list to ensure that it is complete and correct. If you have cited papers that have been retracted, please include the rationale for doing so in the manuscript text, or remove these references and replace them with relevant current references. Any changes to the reference list should be mentioned in the rebuttal letter that accompanies your revised manuscript. If you need to cite a retracted article, indicate the article’s retracted status in the References list and also include a citation and full reference for the retraction notice.

**Response**: Thank you very much for the comments and concerns you raised about the reference list. We have not cited or removed retracted papers. In general we have not changed the reference list except we change the reference formatting to be in line with PLOS one referencing style.

**Additional Editorial comments**

**Comments**: Thank you for resubmitting this paper. I am aware that you have addressed to most of the reviewers' concerns already. However, an additional round of English copyediting work is required, for example in the Abstract section; it is quite awkward to mention "08 Key informant interviews", and"03 focus group" in the main text. Another critical issue is that given the number of participants is quite low, I am afraid some of the participants could be potentially identified by having their information provided in "Table 2" and also other parts of the paper. Please consider how their privacy could be protected in this regard.

**Response**: Thank you very much for appreciating and acknowledging our work. We duly agree with your constructive comments and we did it. For the additional round of English language copy editing we did it with language expert. We rephrased and incorporated the comments raised in the abstract section of the manuscript. The number of the participants was based on the saturation of the data (participants’ descriptions become repetitive).i.e. we continued sampling the participants until no new information emerged and saturation was reached. During designing the data collection, for example, we had planned to conduct four FGDs (two FGD from each district) in addition to the IDIs and KIIs, however, when no new idea was sought from the participants we conducted three FGDs. We have also included different participants to triangulate the data.

Regarding to the privacy of the participants: Thank you very much for your critical observation that some of the participants could be potentially identified by having their information provided in "Table 2”. We found that health extension program coordinators could be identified, because, in each district there is only one health extension program coordinator. Thus, we changed health extension program (HEP) coordinator to district level healthcare authority, as HEP coordinator is a member of district level healthcare authority. We also omitted some of the characteristics of the participants from table “Table 2”. Other participants could not identify (for example: health extension worker supervisors could not identified, because there are many health centers in each district).

In general, participants were de-identified throughout the transcription to ensure the confidentiality and anonymity of the participants. During audio recording, the file names were de-identified using codes and were deleted after transferring from recorder to personal protective device. Personal identifiers were also omitted during writing the description. Confidentiality was strictly maintained throughout the study and only the researchers have access to the data. No personal identifying information was retained that could identify the participants. Confidentiality was also practiced at every stage including anonymising the quotes from the interviews.

We again thank the editors and reviewers for their insightful and constructive comments.
