# Supplementary material for: “Mothers will be lucky if utmost receive a single scheduled postnatal home visit”: An exploratory qualitative study, Northern Ethiopia
Source: PLoS One. 2022 Mar 30;17(3):e0265301. doi: 10.1371/journal.pone.0265301 (PMC8967047; doi:10.1371/journal.pone.0265301)
Supplement: Attachment — Submitted filename: Reviewer Comments.docx [file pone.0265301.s006.docx]

To

The Editor

PLOS ONE

Dear Editor,

Thank you for the opportunity to review this manuscript. The manuscript is well written and appreciate the hard work involved in the conduct of the study

Please see inputs section-wise below

**Material and method**

Suggest to follow the COREQ guideline [http://www.ijo.in/documents/05COREQ SS.pdf](http://www.ijo.in/documents/05COREQ%20SS.pdf)

1. Please mention the research team and their credentials, who conducted qualitative interviews and FGDs, experience, and training.
2. Study design should include

- The theoretical framework used (methodological orientation) (e.g., grounded theory, discourse analysis, ethnography, phenomenology, content analysis)
- Participant selection: sampling, method of approach, reasons for refusals (if any), any experience/challenges in selecting participants for focus group discussion, how to overcome those challenges (can also be discussed in the discussion section)
- In the data collection section, mention any repeat interview (if any) and reason for the same. Please upload the Interview guide as supplementary material.

3. Data analysis: how the member checking was conducted (at present the sentence seems to be incomplete)

**Result**

Suggestion to reduce the quotable quotes or present the quotes in a tabular format theme-wise

**Discussion**

o Editing is required to avoid repetition from the result section

o What’s new in this study? Why should this article be cited? What is that ‘X’ factor?

o How the barriers (home visits) in Africa or your region is different from other low and middle-income countries?

o What significant steps were taken by the other African countries or low and middle-income countries to address barriers

o How the findings could be used: application/ translation and way forward. Recommendation to the government to address the barriers

Manuscript presented in an intelligible fashion: Yes however, **text and language editing is required**

Thanks

Best wishes

Narendra K Arora
